# Visualization of Phosphatidic Acid Fluctuations in the Plasma Membrane of Living Cells

**DOI:** 10.1371/journal.pone.0102526

**Published:** 2014-07-15

**Authors:** José P. Ferraz-Nogueira, F. Javier Díez-Guerra, Juan Llopis

**Affiliations:** 1 Centro Regional de Investigaciones Biomédicas and Facultad de Medicina de Albacete, Universidad de Castilla-La Mancha, Albacete, Spain,; 2 Centro de Biología Molecular Severo Ochoa and Departamento de Biología Molecular, Facultad de Ciencias, Universidad Autónoma de Madrid, Madrid, Spain; UPR 3212 CNRS -Université de Strasbourg, France

## Abstract

We developed genetically-encoded fluorescent sensors based on Förster Resonance Energy Transfer to monitor phosphatidic acid (PA) fluctuations in the plasma membrane using Spo20 as PA-binding motif. Basal PA levels and phospholipase D activity varied in different cell types. In addition, stimuli that activate PA phosphatases, leading to lower PA levels, increased lamellipodia and filopodia formation. Lower PA levels were observed in the leading edge than in the trailing edge of migrating HeLa cells. In MSC80 and OLN93 cells, which are stable cell lines derived from Schwann cells and oligodendrocytes, respectively, a higher ratio of diacylglycerol to PA levels was demonstrated in the membrane processes involved in myelination, compared to the cell body. We propose that the PA sensors reported here are valuable tools to unveil the role of PA in a variety of intracellular signaling pathways.

## Introduction

Phosphatidic acid (PA) is an acidic phospholipid that plays a central role in the biosynthesis of other lipids. By serving as a substrate or by modulating the activity of various enzymes, it participates in the complex network of structural, energy storage, and signaling lipids [1]. Using phosphatidylcholine as a substrate, PA can be synthesized by phospholipase D (PLD), and converted into diacylglycerol (DAG) by PA phosphatases. DAG can be converted back into PA by DAG kinases (DGK). Moreover, PA can be metabolized by phospholipase A_2_ to generate lysophosphatidic acid (LPA), whereas the reverse reaction is catalyzed by lysophosphatidic acid acyl transferases [1]–[3] (see Fig. S1). In addition, PA itself is a lipid mediator [3], and its growing list of effector molecules includes proteins involved in cytoskeleton rearrangement, vesicle trafficking, cell growth, spreading, proliferation, and survival [2], [3]. Importantly, with the exception of PLDs, the above mentioned enzymes either render or metabolize another signaling lipid, thus exerting a signaling-switch activity between PA and other pathways. Moreover, PA is a small cone-shaped phospholipid that provides flexibility to cellular membranes. It stabilizes the negative curvature of lipid bilayers, helping in the formation of vesicles from Golgi apparatus or plasma membrane [4], and mediating fusion and fission events of organelles such as mitochondria [5].

Traditionally, PA levels have been measured using thin-layer chromatography or liquid chromatography coupled to mass spectrometry [6], [7]. However, these techniques do not provide the desired spatio-temporal resolution for some applications. Further, variations in the signaling pools of PA are often obscured by larger PA pools involved in intermediary metabolism (for example, in the endoplasmic reticulum). To reveal PA production at the cellular and subcellular levels, several biosensors featuring PA-binding domains (PABD) attached to fluorescent proteins have been reported [8]–[11]. Such probes relying on membrane translocation and a single fluorescence signal do not discriminate between real PA rises and changes in the thickness of the cell or membrane ruffling events, which would also affect fluorescence [12]. In addition, translocation sensors cannot be targeted, hampering the study of PA fluctuations in specific subcellular compartments.

In the present work, we have developed FRET sensors to monitor PA dynamics in the plasma membrane using the PA-binding domain (PABD) of the yeast protein Spo20 (residues 51–91) [9]. We found an inverse relation between plasma membrane PA levels and the FRET efficiency of the sensor. Interestingly, the studies carried out with the sensor indicated a redistribution of PA between the leading and trailing edges of migrating cells. In cells derived from oligodendrocytes and Schwann cells, PA levels were higher in the cell body than in the membrane processes involved in myelination. In contrast, DAG levels were lower in the cell body than in these membrane processes.

## Results

### Construction of a plasma membrane PA sensor based on the yeast SNARE protein Spo20

**Figure 1:**
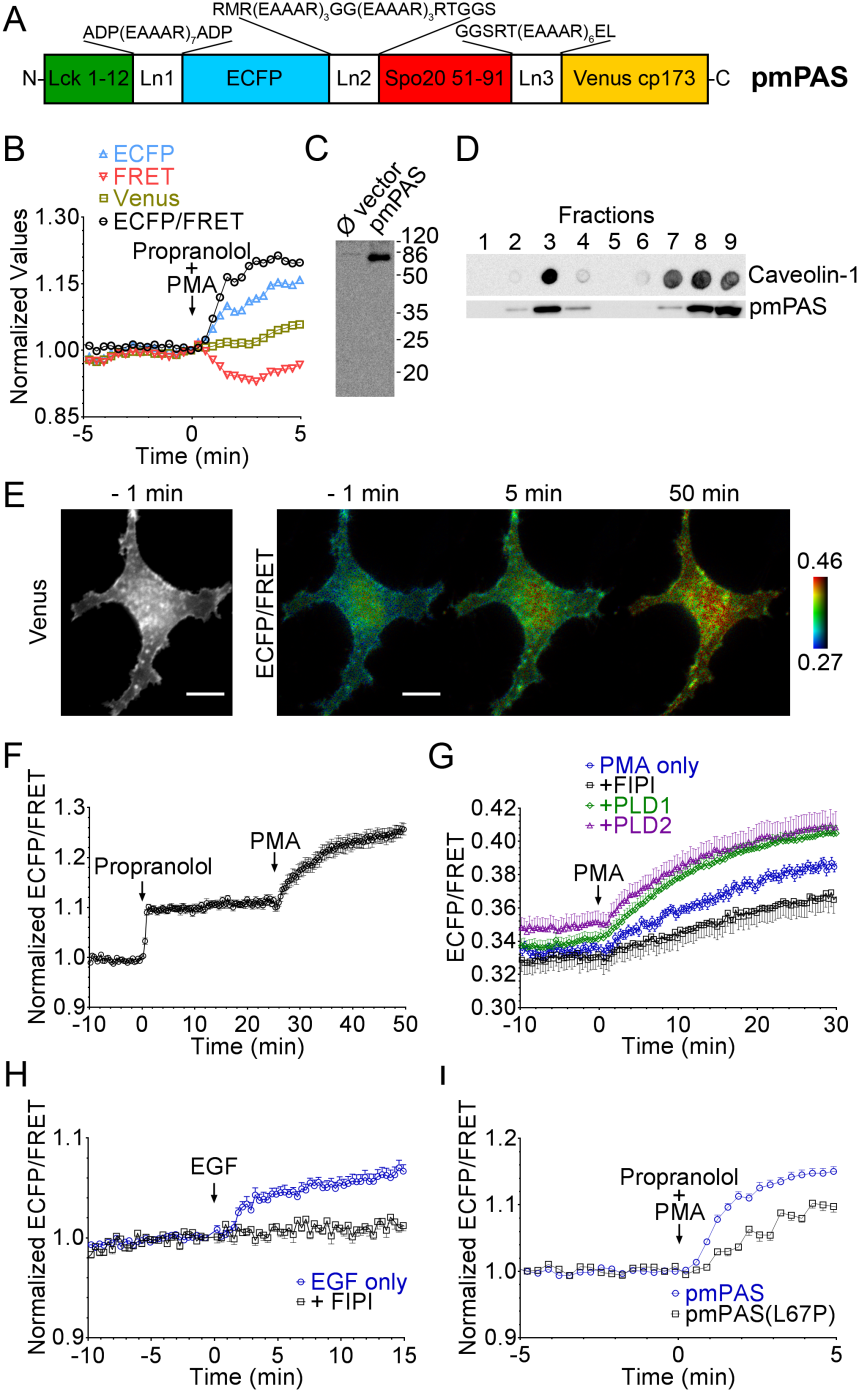
Structure and characterization of pmPAS, a plasma membrane FRET biosensor for PA. (**A**) Structure of the chimera: Lck, lymphocyte-specific protein tyrosine kinase; Ln, linker. (**B**) Time course of fluorescence intensities of ECFP, Venus and FRET imaging channels, and ECFP/FRET ratio (all normalized to time 0), in a representative cell expressing pmPAS and challenged with propranolol 100 µM plus PMA 100 nM. (**C**) Extracts of cells transfected with empty vector or pmPAS were run in SDS-PAGE gels and immunoblotted with anti-GFP antibody. (**D**) Cells expressing pmPAS were lysed and subjected to sucrose gradient ultracentrifugation to check the localization of pmPAS in subdomains of the plasma membrane. Fractions were probed for GFP (Western blot) or caveolin-1 (dot blot). (**E**) Venus fluorescence and pseudocolor ratio images (ECFP/FRET channels) of a representative HeLa cell expressing pmPAS stimulated sequentially with propranolol (100 µM) and PMA (100 nM). Scale bars represent 20 µm and the ECFP/FRET images were coded according to the indicated pseudocolor scale. (**F**) Time course of normalized ECFP/FRET values in regions of interest on the plasma membrane of cells stimulated as in (E) (*n* = 8, 3 independent experiments). (**G**) pmPAS expressing cells were stimulated with PMA (100 nM) with or without preincubation with FIPI (1 µM, 30 min) (*n* = 9, 4 independent experiments for each condition). Cells coexpressing PLD1 (*n* = 7, 3 independent experiments) or PLD2 (*n* = 8, 3 independent experiments) and pmPAS were also stimulated with PMA. (**H**) HeLa cells expressing pmPAS were stimulated with EGF (100 ng/ml) only or preincubated with FIPI (1 µM, 30 min) (*n* = 7 for EGF only, *n* = 6 for preincubation with FIPI, 3 independent experiments for each condition). (**I**) HeLa cells expressing pmPAS or pmPAS(L67P) were challenged with 100 µM propranolol and 100 nM PMA (*n* = 12 and 4 independent experiments for pmPAS, *n* = 9 and 3 independent experiments for pmPAS(L67P)). Error bars indicate the mean±SEM.

Several PABDs have been described with sufficient specificity to be used in fluorescent PA reporters [8]–[11]. We attempted to construct a FRET biosensor using the small synaptic protein neurogranin [13] as the PABD. However, stimuli leading to PA production in the plasma membrane failed to elicit FRET changes in several variants of chimeras featuring non-calmodulin binding neurogranin mutants. Therefore, we turned to the yeast SNARE protein Spo20, which has been shown to bind specifically to PA *in vivo* [9]. Previously, fusion of Spo20 residues 51–91 and GFP has been used to create translocation sensors for PA [8], [11], [14]. Hence, we used this Spo20 PABD to construct FRET-based sensors for reporting PA changes in the plasma membrane. The sensors consist of a plasma membrane targeting sequence (residues 1–12 of Lck) [15], ECFP as the donor fluorescent protein, residues 51–91 of Spo20 as the PABD, and Venus as the FRET acceptor (Fig. 1A). Five circular permutations of Venus (cpVenus), with their structure starting at different points in the original sequence [16], were also employed to create chimeras showing variability in donor-acceptor orientation and distance. To allow the necessary mobility of domains, three linkers of 37–41 residues, based on repetitions of the pentapeptide Glu-Ala-Ala-Ala-Arg, were introduced in between them. A similar approach was used previously for the generation of FRET indicators for DAG [17] and phosphatidylinositols [18].

Phorbol esters increase PLD activity via activation of PKC [4], [19], whereas propranolol, acting on PA phosphatases, rapidly inhibits PA conversion to DAG [20]. Thus, phorbol 12-myristate 13-acetate (PMA) and propranolol added together are a strong stimulus to increase cellular PA levels. Upon perfusion of HeLa cells expressing the chimeras with PMA (100 nM) and propranolol (100 µM), fluorescence decreased in the FRET channel and increased in the donor ECFP channel (less FRET, Fig. 1B) in all chimeras; thus, higher ECFP/FRET ratios indicate higher PA levels. The chimera that showed the lowest basal ECFP/FRET ratio (0.31, Fig. S2A) and the largest ratio increase upon stimulation (Fig. S2B) contains a circularly-permuted Venus on residue 173. It was named pmPAS (*p*lasma *m*embrane *PA S*ensor) and used in subsequent experiments. We also constructed a chimera containing two copies of Spo20 (51–91), but the ratio change obtained by raising PA levels was smaller than that of pmPAS (Fig. S2C). Confocal microscopy confirmed the localization of pmPAS in the plasma membrane (Fig. S2D).

We imaged HeLa cells expressing pmPAS and used the 3-cube method (see Materials and Methods) to unmix the fluorescence images, and to obtain ECFP (donor), cpVenus173 (acceptor) and FRET images without spectral crosstalk (Fig. S3A). This method also allowed calculation of donor to acceptor expression ([D]/[A]) images and FRET efficiency (*E*) images (Fig. S3B). In all pmPAS cells, [D]/[A] was found to be 1, showing equimolar amounts of ECFP and cpVenus173, as expected. After raising PA levels with PMA plus propranolol, the FRET efficiency *E* decreased, whereas the [D]/[A] did not change. Furthermore, extracts of cells expressing the chimera showed a unique anti-GFP reactive band of the expected size in Western blots (Fig. 1C), which demonstrated the stability of the chimera in the cellular environment.

The N-terminal tail of Lck is myristoylated and palmitoylated, and has been shown to be sufficient to anchor recombinant proteins to the plasma membrane [21]. Such combination of acylations should also favor anchoring the biosensor to lipid rafts, where signaling molecules such as PLD have been shown to organize into functional complexes [22]. Sucrose gradient ultracentrifugation of post-nuclear cell extracts showed that pmPAS colocalized with caveolin-1, a marker of lipid rafts, in low density fractions (Fig. 1D), but both labels were also present at higher densities. Therefore, pmPAS should be sensitive to changes of PA in both lipid raft and non-raft domains of the plasma membrane.

### Modulation of PLD activity affects pmPAS response

The response to PA-phosphatase inhibition by propranolol was within seconds of addition, suggesting a fast and dynamic turnover of PA in HeLa cells (Fig. 1E–F). Further stimulation with PMA caused the ECFP/FRET ratio to increase even more, reaching a steady state after 25 min (Fig. 1F, Video S1). Overexpression of PLD1 or PLD2 by cotransfection with pmPAS enhanced the PMA response (Fig. 1G); PLD2 slightly increased the basal PA levels (basal ECFP/FRET); in fact, overexpressed PLD2 is known to be active without further stimulus. On the other hand, preincubation with the PLD1/PLD2 inhibitor 5-fluoro-2-indolyl des-chlorohalopemide (FIPI, 1 µM) decreased the rate and magnitude of the PMA response, but did not abolish the ratio change (Fig. 1G). Since PLDs are completely inhibited at that concentration of FIPI [23], the PMA and PKC activation may be having effects on PLC or DAG kinase to generate PA. The effect of FIPI was confirmed using the PA-translocation sensor GFP-Spo20 [8]. This probe exhibited a predominant localization in the cell nucleus, but upon addition of PMA it accumulated in the plasma membrane (Fig. S4). Physiological stimulation of EGF receptors has been shown to activate PLD and increase PA levels in the plasma membrane [4], [24]. Using serum-starved HeLa cells expressing pmPAS, the addition of EGF resulted in an increase of ECFP/FRET (a PA increase), which was abrogated by preincubation with FIPI (Fig. 1H), suggesting the involvement of PLD activity.

It is predicted that the PA binding domain of Spo20 forms an amphypathic alpha-helix, with hydrophobic and positively charged faces. It was shown that the substitution in the amphypathic face of Pro for Leu67 (mutation L67P) diminishes the affinity of this domain for PA, probably by breaking the helix structure [9]. Indeed, this seems to be the case, since we observed that basal FRET was lower in a modified pmPAS with mutation L67P in its Spo20 domain, which is consistent with increased distance or altered chromophore orientation. Moreover, the response of this mutated chimera to propranolol plus PMA was three-fold slower than control pmPAS (0.026 and 0.088 ratio units/min, respectively; Fig. 1I), suggesting that the L67P biosensor had lower affinity for PA.

### Stimulation of pmPAS expressing cells with PA and oleic acid liposomes

**Figure 2:**
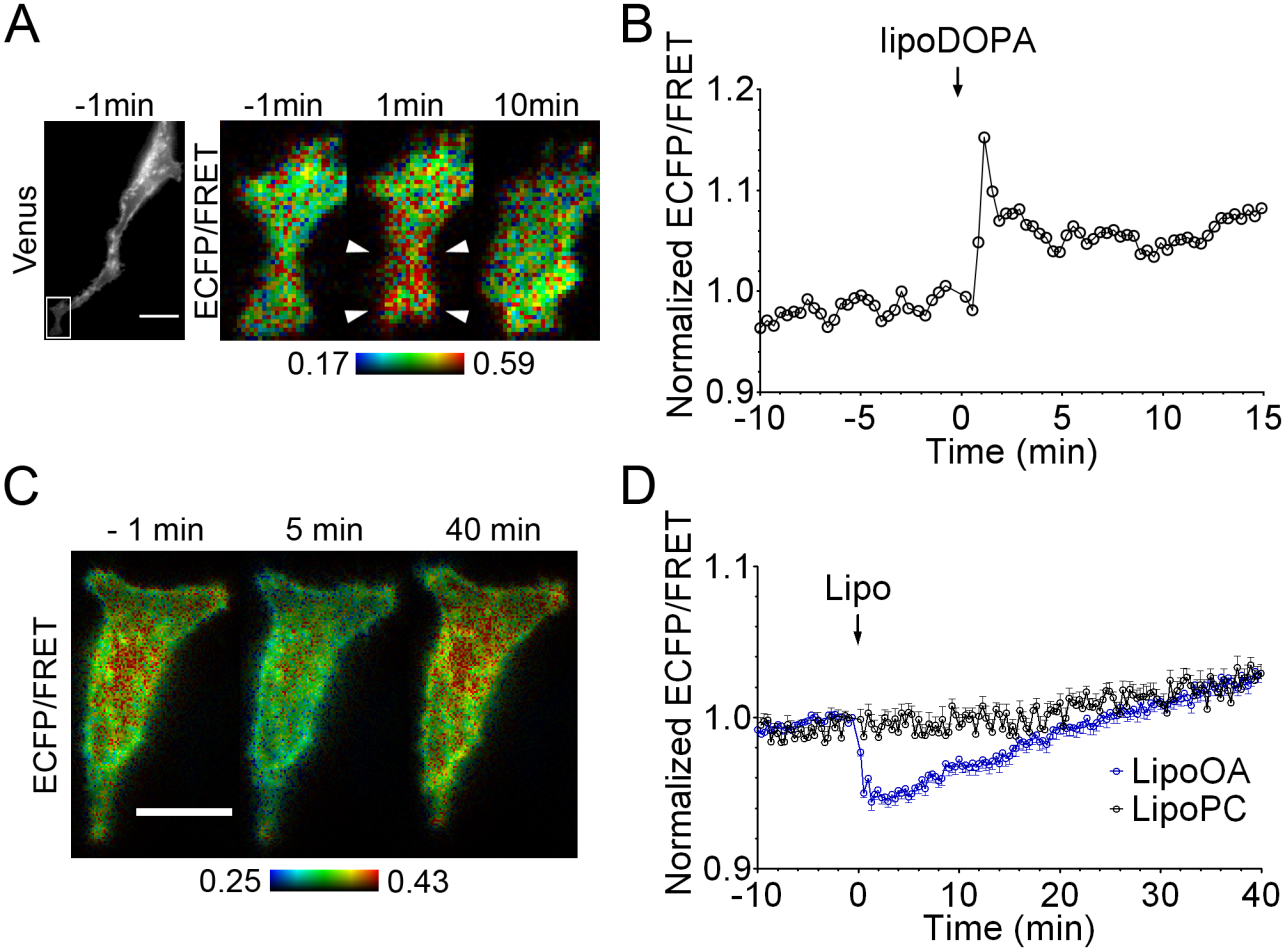
Response of pmPAS to liposomes containing dioleoylphosphatidic acid or oleic acid. (**A**) Fluorescence intensity (Venus channel) and ECFP/FRET ratio images of a HeLa cell expressing pmPAS challenged with liposomes containing dioleoylphosphatidic acid (lipoDOPA, 200 µM). (**B**) Time course of normalized ECFP/FRET of the cell shown in (A). The time course and magnified images refer to the area indicated by a box in the gray image. (**C**) ECFP/FRET ratio images of a HeLa cell expressing pmPAS at the indicated time (min) before or after addition of liposomes containing oleic acid (lipoOA, 500 µM). (**D**) Time course of normalized ECFP/FRET of cells challenged with lipoOA (as in (C)) (the cell average of 4 cells is shown), or incubated with phosphatidylcholine liposomes (lipoPC) (*n* = 6, 3 independent experiments). Error bars indicate the mean±SEM. Scale bars represent 20 µm and the ECFP/FRET images were coded according to the indicated pseudocolor scale.

Another way to characterize the response of the indicator to PA, besides the physiological or pharmacological manipulations described above, was to stimulate cells directly with the phospholipid. PA cannot be directly added to the cells due to its insolubility in water. Instead, suspensions containing PA were subjected to a protocol to produce liposomes (see Materials and Methods), which are able to fuse with the plasma membrane [25]. We challenged HeLa cells expressing pmPAS with liposomes containing dioleoyl phosphatidic acid (lipoDOPA). Addition of lipoDOPA (200 µM) resulted in a rapid increase of ECFP/FRET followed by a partial recovery within minutes (Fig. 2A–B and Video S2), confirming the responsiveness of pmPAS to exogenously added PA. The response to lipoDOPA varied from cell to cell; these variations in intensity and time course may be due to heterogeneity in liposome size and fusion with cells. Representative cells expressing pmPAS are shown in Fig. S5A–B to illustrate this variability. The effect of lipoDOPA was also studied in HeLa cells expressing the translocation sensor GFP-Spo20. Plasma membrane fluorescence increased upon stimulation with lipoDOPA, also showing some variability of intensity and time course (a representative cell is shown in Fig. S5C).

Several reports indicate that oleic acid (OA) activates lipins, phosphatases that convert PA to DAG, by inducing their translocation to the plasma membrane [26]; thus, OA should decrease PA levels, the opposite effect of propranolol. Addition of liposomes containing phosphatidylcholine and oleic acid (lipoOA, 500 µM) resulted in a rapid decrease of ECFP/FRET, followed by a slow recovery (Fig. 2C–D and Video S3). No effect was seen with liposomes containing phosphatidylcholine alone (Fig. 2D). As for lipoDOPA, variations in intensity and time-course were observed from cell to cell (Fig. S6).

Since PA can be converted to the signaling lipid LPA by the action of phospholipase A2, we investigated the possibility that the effects of PA increase on pmPAS fluorescence could be due to LPA. We challenged MSC80 cells expressing pmPAS with LPA incorporated in phosphatidylcholine liposomes. The ECFP/FRET ratios did not increase, suggesting that the effects of pmPAS in response to PA-increasing drugs, or liposomes carrying PA, described so far, were not due to the conversion of PA to LPA.

Interestingly, the ECFP/FRET ratios decreased after incubation with LPA liposomes (Fig. S7A and D), whereas control phosphatidylcholine liposomes showed no effect (Fig. S7B and D). This could be explained if stimulation with LPA decreased PA content at the plasma membrane. Addition of LPA alone to the cells (not incorporated in liposomes) resulted in an ECFP/FRET ratio decrease (Fig. S7C and D), but this treatment also caused rapid changes in cell shape. Further experiments will have to be conducted to find out whether these effects are mediated by LPA receptors and which enzymes are involved.

Summing up the previous results, PA binding to the sensing moiety Spo20 resulted in a decrease of resonance energy transfer between the two fluorescent proteins incorporated into the biosensor pmPAS. The response was reversible, and pmPAS reported ECFP/FRET ratio changes upon fluctuations in cell PA levels. Stimuli that increase PA levels (PMA, propranolol, EGF and lipoDOPA) always increased ECFP/FRET ratio, whereas lipoOA, which decreases PA levels, decreased it. Moreover, the introduction of a point mutation in the Spo20 domain conferring lower affinity for PA slowed down the response of pmPAS to PA. Taken together, these results indicate that the changes in ECFP/FRET ratio of pmPAS are due to a specific interaction of the probe with PA.

### Difference in plasma membrane PA levels in various cancer cell lines

**Figure 3:**
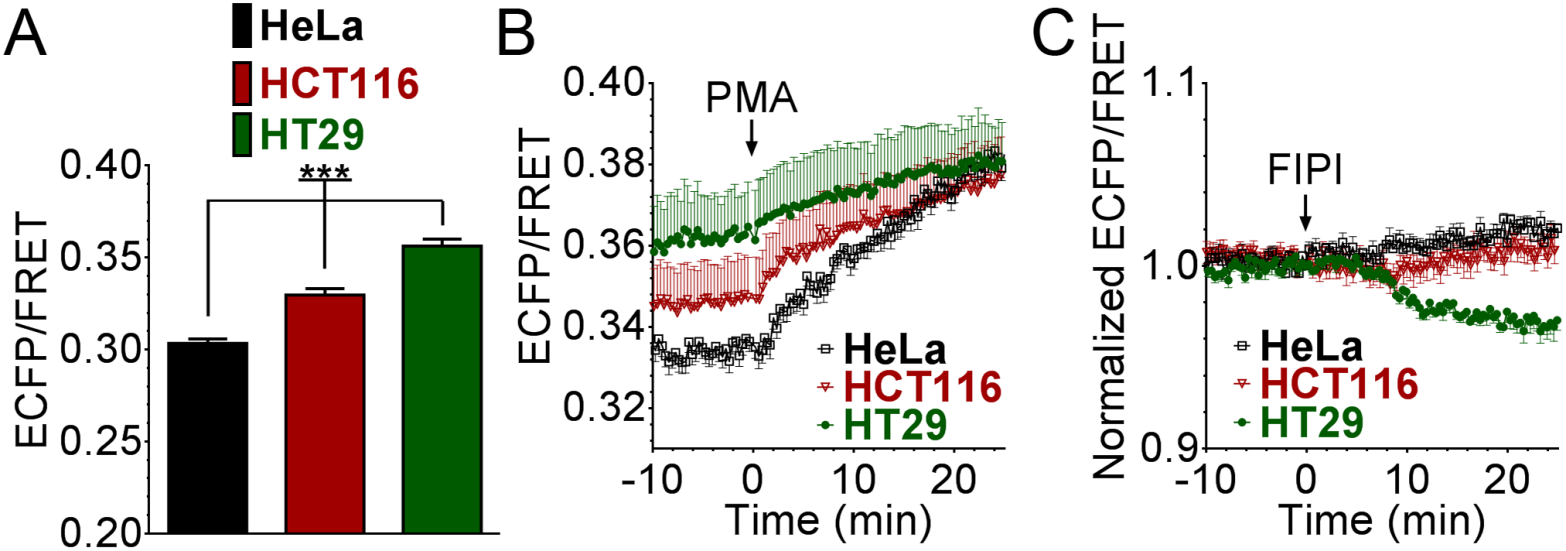
Variability of plasma membrane PA levels in various cancer cell lines. HeLa, HCT116 and HT29 cells were transiently transfected with pmPAS. (**A**) Basal ECFP/FRET ratios on regions of interest on the plasma membrane were determined (HeLa *n* = 45, HT29 *n* = 48, HCT116 *n* = 53, ***p<0.0001 using one-way ANOVA). (**B**) Time course of ECFP/FRET values of cells in response to PMA (100 nM) (HT29: *n* = 7, 3 independent experiments; HCT116: *n* = 6, 3 independent experiments; HeLa: *n* = 9, 4 independent experiments) (**C**) Time course of normalized ECFP/FRET values of cells in response to FIPI (1 µM) (HT29: *n* = 6, 4 independent experiments; HCT116: *n* = 8, 4 independent experiments; HeLa: *n* = 9, 4 independent experiments), respectively. Error bars indicate the mean±SEM.

We used pmPAS to compare the basal levels of PA in the plasma membrane of HeLa cells, derived from a human cervical adenocarcinoma, and the human colon cancer-derived cell lines HT29 and HCT116, two cell types with intermediate and low capacity to differentiate, respectively; HCT116 cells have high clonogenic and tumorigenic potential. As shown in Fig. 3A, HeLa cells exhibited the lowest levels of PA and HT29 cells the highest. Interestingly, upon addition of PMA to these cells, the PA levels rose to a similar value in the three cell types (Fig. 3B), suggesting that HT29 cells had higher basal PLD activity than HeLa cells. Consistent with this, PA levels in HT29 cells decreased after adding the PLD inhibitor FIPI, whereas HeLa and HCT116 cells did not show any change (Fig. 3C). This suggests that indeed a PLD1/2 activity is present in the plasma membrane of unstimulated HT29 cells. In fact, PLD activity has been reported to be elevated in lipid rafts of multidrug resistant HT29 cells [27]. In addition, increased PLD activity has been reported in several types of human cancers [24], [28]. We conclude that pmPAS may serve to characterize PA levels in the plasma membrane of cancer cells.

### Plasma membrane PA gradients in polarized cells

Live cells in culture are seen to emit filopodia and lamellipodia, highly dynamic actin structures that result from rearrangement of the actin cytoskeleton. Both structures play a role in cell polarization, morphology, migration, cell-cell and cell-extracellular matrix adhesion, all critical events for processes such as cancer cell invasion or neuronal polarization [29]–[31]. In cells expressing a plasma membrane-anchored fluorescent protein such as pmPAS, these structures are easily identified, thus HeLa cells expressing pmPAS were seen to randomly emit filopodia and lamellipodia.

Interestingly, upon addition of propranolol to HeLa cells, lamellipodia and filopodia activity slowed down (Video S1), whereas liposomes containing oleic acid increased membrane dynamics at the edges (Video S3). These results suggest an inverse relationship between the PA levels and motility of the plasma membrane, which is consistent with previous studies reporting accumulation of DAG at the leading edge of migrating cells [32], since the levels of these lipids are balanced by the net activity of DGK and PAP. This prompted us to explore potential changes of PA levels associated with membrane motility.

**Figure 4:**
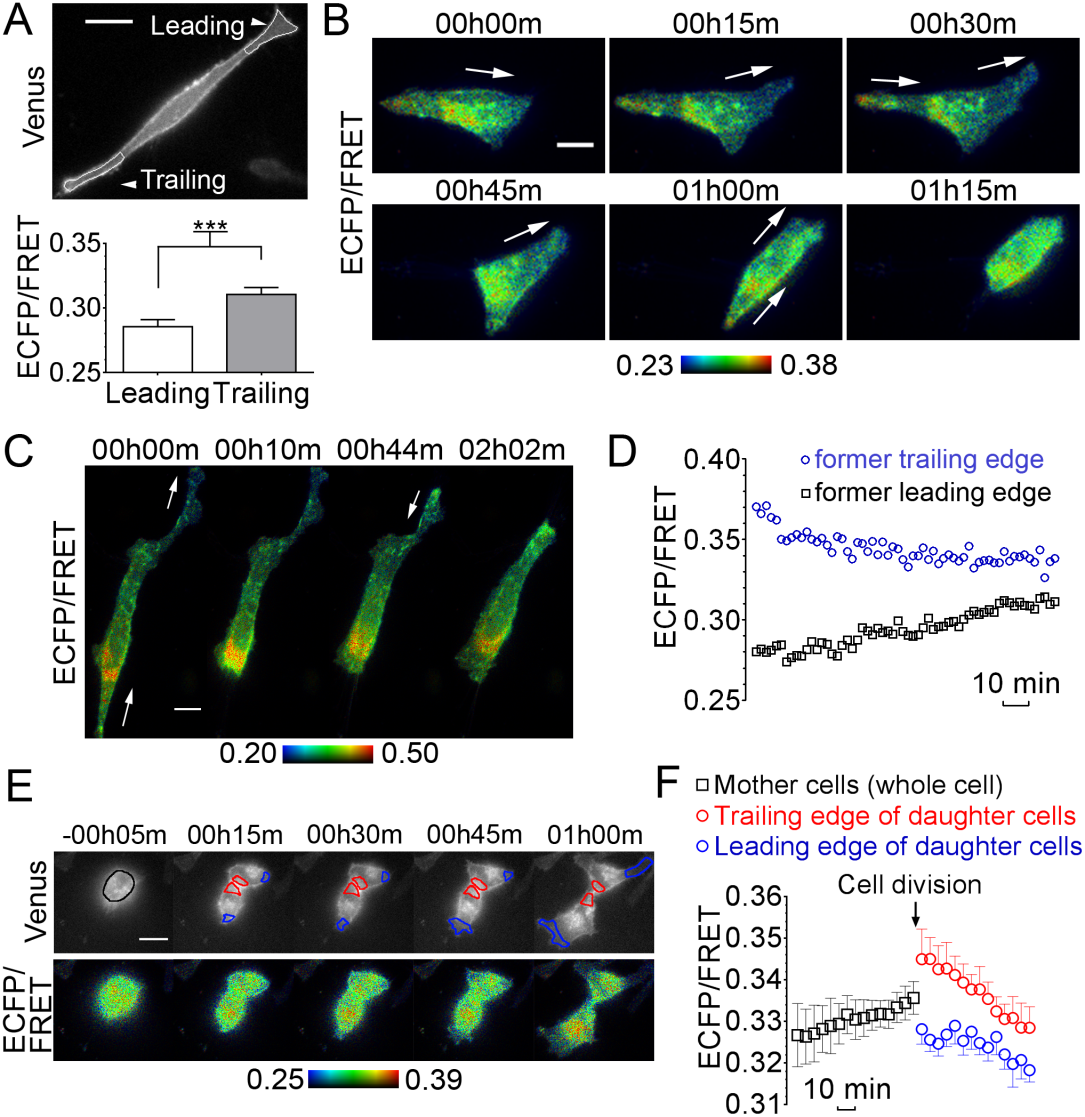
PA gradients in migrating cells and during cell division. (**A**) A polarized HeLa cell with leading and trailing edges is shown. Average ECFP/FRET ratio in the leading and trailing edges of polarized HeLa cells (*n* = 18, p<0.01 using *t*-test). (**B**) and (**C**) Timelapse of pseudocolor ratio images (ECFP/FRET) of migrating HeLa cells expressing pmPAS. Cells were kept at 37°C, 5% CO_2_ in fully supplemented medium under the microscope. (**D**) Time course of ECFP/FRET values of the cell represented in (C). (**E**) Timelapse (intensity and pseudocolor ratio images) of a cell expressing pmPAS during mitosis. (**F**) ECFP/FRET values of 4 cells during mitosis (4 independent assays). The ECFP/FRET ratio was measured in regions of interest as indicated in (E); blue and red code for regions at the leading edge and the abscission zone of the daughter cells, respectively. Scale bars represent 20 µm and arrows show the direction of cell movement. Error bars indicate the mean±SEM.

In HeLa cells transfected with pmPAS cultured at low density it was not difficult to observe cells showing sustained directed migration. In these cells, we found higher PA levels at the plasma membrane of the trailing edge than the leading edge (Fig. 4A, Video S4). Fig. 4B shows timelapse images of a cell migrating to the right and upward; again, higher PA levels were observed at the trailing edge, and this difference was maintained while cells remained motile. A reduction of this PA gradient was observed in cells retracting the leading edge (Fig. 4C–D, Video S5).

During these experiments, some cells undergoing mitosis were imaged. Before cell division, cells round up and partially detach from the dish surface. Consistent with our previous findings, plasma membrane PA levels were lower at the leading edge of the dividing cells, where they spread out on the dish, than at the area next to the abscission site (Fig. 4E–F).

### Heterogeneity of plasma membrane PA levels in myelinating cells

Myelination is a key process in the development of the vertebrate nervous system. Through molecular mechanisms that are still poorly understood, oligodendrocytes (in the central nervous system) and Schwann cells (in the peripheral nervous system) extend membrane processes from the cell body to establish long-lasting contacts with axons; they wrap the axons with several membrane layers and produce a myelin sheath. The extension and targeting of these processes is critical for proper myelination [33], and the role of lipid signaling in this context is poorly understood.

**Figure 5:**
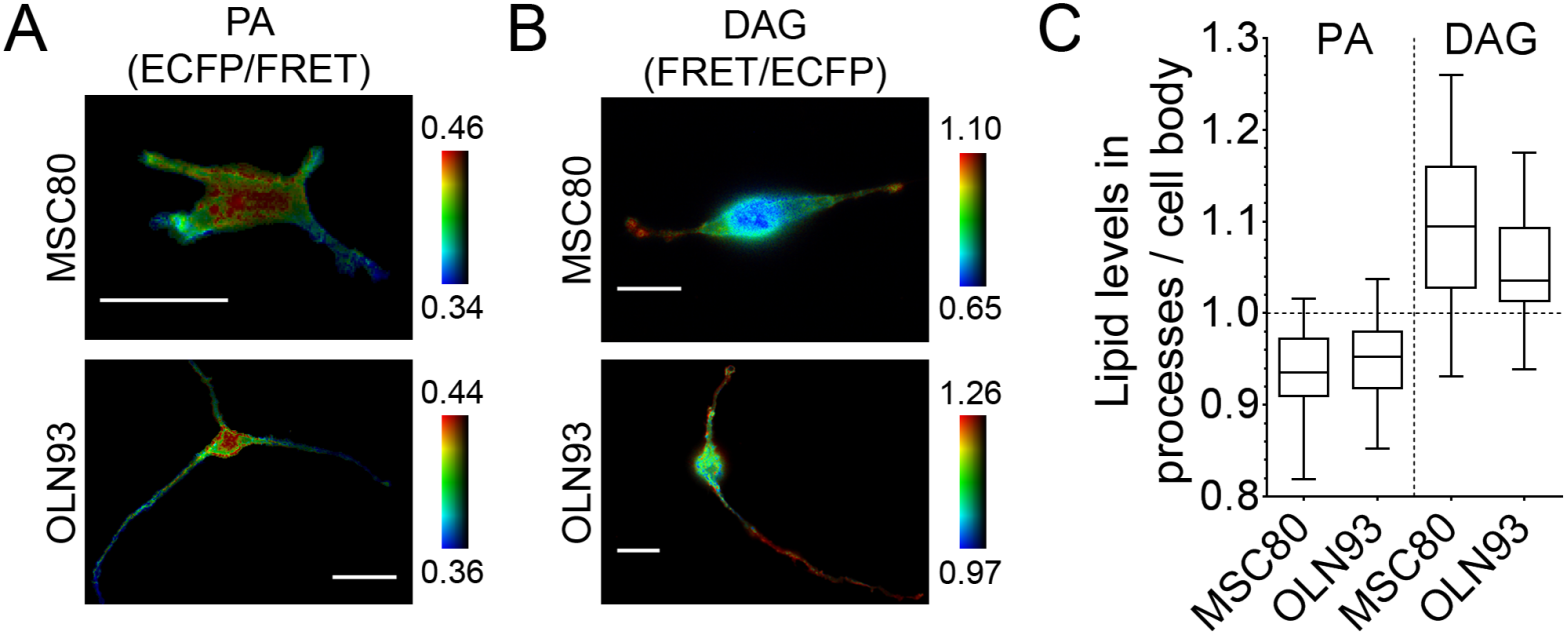
PA and DAG gradients in MSC80 and OLN93 cells. (**A**) Pseudocolor ratio images (ECFP/FRET) of representative cells expressing pmPAS. (**B**) Pseudocolor ratio images (FRET/ECFP) of representative cells expressing Daglas-pm1. Scale bars represent 20 µm. The ratio images were coded according to the indicated pseudocolor scale. (**C**) Quantitative analysis of MSC80 and OLN93 cells expressing either pmPAS or Daglas-pm1. Ratios of cell processes were averaged and compared with ratios of the cell body. Values obtained represent the process:cell body fold difference. The middle line in the Box-and-Whisker plot represents the median, the central box the values from 25th to 75th percentile, and the bars reach 1st-99th percentile (pmPAS: MSC80 *n* = 58 cells, OLN93 *n* = 64 cells; Daglas-pm1: MSC80 *n* = 23 cells, OLN93 *n* = 36 cells).

To analyse if PA might be involved in such processes we followed the changes in ECFP/FRET ratios of the mouse cell lines MSC80 (Schwann cells) and OLN93 (oligodendrocytes) expressing pmPAS. As shown in Fig. 5A and C, PA levels were lower in the processes than in the cell body of both cell types. Since previous studies have shown that DAG levels are higher at the leading edge of migrating cells [32], it was interesting to check DAG levels in MSC80 and OLN93 cells. We expressed Daglas-pm1, a FRET probe for DAG [17], in the plasma membrane of MSC80 and OLN93 cells and compared the FRET ratio in the processes and cell body. DAG levels were higher in the processes than in the cell body (Fig. 5B and C). We suggest that a higher ratio of PA to DAG levels in the cell body would prevent growth of new processes whereas a lower ratio of PA to DAG levels in the processes would stabilize them and allow their extension.

## Discussion

A recent advance on PA indicators based on translocation has been the addition of a nuclear export signal to the single intensity sensor GFP-Spo20, which eliminated nuclear staining and increased the probe sensitivity [34]. However, fluorescent sensors based on ratio imaging have some intrinsic advantages over single intensity indicators. For instance, the effects of probe concentration or photobleaching cancel out when two images are divided to yield a ratio. Also, FRET sensors for signaling lipids can be targeted to specific cellular subdomains to measure their local changes. In the case of PA, small local variations may be hard to discern with translocation sensors, since large PA pools related to triacylglycerol metabolism exist. A previously reported FRET-based sensor for PA detection in the plasma membrane, termed Pii, revealed the heterogeneity in level and distribution of PA in single cells [20]. A fragment of human DOCK2 was used as the PABD, and it was targeted to non-raft membrane domains with a K-Ras4B tag. In contrast to Pii, pmPAS should also be sensitive to PA changes in lipid rafts. Moreover, pmPAS was sensitive to both rises and decreases in PA; this has not been shown with either translocation sensors or Pii.

In pmPAS, engagement of the lipid binding domain to the PA target decreased FRET, in contrast with sensors for PI(3,4,5)P_3_ [35] or DAG [17], [32], in which binding of the lipid to the chimera resulted in increased FRET. Without structural data, it is hard to predict whether FRET will increase or decrease in response to lipid binding in these chimeric proteins. It is interesting to note that, in the previously reported probes Pii-DK and Pii-SS [20], FRET decreased in response to PA, despite the fact that the PA binding motifs used in these probes (DOCK-2, SoS1) are unrelated to Spo20, used here in pmPAS.

Why would FRET in pmPAS decrease upon binding PA? We speculate that, in the absence of PA, binding of the polycationic alpha helix of Spo20 (51–91) to negatively charged residues in the linkers of pmPAS could favor a folded biosensor, with less distance between ECFP and cp173Venus, whereas binding of Spo20 (51–91) to PA on the plasma membrane would result in a conformation less suitable for FRET, due to an increase of donor-acceptor distance, a change of the relative orientation of the fluorochromes, or a combination thereof.

In this study, of the various YFP Venus variants tested, cp173Venus resulted in the strongest basal FRET (Fig. S2A) and largest ratio change with PA-increasing stimuli (Fig. S2B). Circular permutations of Venus were used in the Ca2+ indicators, yellow cameleons, in an attempt to vary the orientation of the FRET acceptor relative to the donor. Nagai and coworkers [36] have proposed an explanation for the fact that cp173Venus resulted in higher FRET efficiency (in the chimera named YC3.60) than Venus or other circular permutations. In some GFP crystal structures, GFP appear as dimers in an anti-parallel configuration [37]. If this tendency to form a weak dimer with antiparallel orientation is conserved in the yellow cameleons or variants of pmPAS of this study, of all available Venus variants, cp173Venus would provide the closest distance between the C-terminus of ECFP and N-terminus of the acceptor. Since, in pmPAS, Spo20 (51–91) is linked to cp173Venus by a rigid alpha helical peptide (linker 3), binding of the Spo20 moiety to membrane PA could disrupt the ECFP:cp173 Venus dimer conformation, resulting in less FRET.

We found an interesting relationship between plasma membrane PA levels and membrane motility in HeLa cells, it decreased with inhibition of PA phosphatase by propranolol, and increased with activation by oleic acid. This suggests that membrane motility is linked to the PA–DAG balance in these cells. In addition, we showed a PA gradient in migrating cells, with higher levels at the trailing edge than at the leading edge (Fig. 4, Video S4). Previous reports have shown higher DAG levels in the leading edge of migrating cells [32]. Hence, a high ratio of DAG/PA levels is characteristic of membrane protrusion in the leading edge, whereas low DAG/PA is associated with retraction of membrane projections. We also showed that PA levels decreased in cells after mitosis, which reattach to the dish and begin to spread (Fig. 4E–F). In agreement with this, it has been reported that non-attached cells have higher PLD2 activity than attached cells. The lower PA levels in early spreading cells (low PLD2 activity) were associated with a higher activity of myosin phosphatase [8], which is inhibited by PA. This phosphatase dephosphorylates myosin light chain, leading to decreased cortical contractile forces and promotion of spreading. Accordingly, HT29 cells, which had high basal PLD activity and PA levels (Fig. 3), displayed a more rounded shape and weaker adhesion to the dish than HCT116 cells (Fig. S8) or HeLa cells.

It is important to highlight that the mechanisms regulating random migration of unstimulated HeLa cells may be different from those modulating other polarized phenotypes. For instance, it has been shown that upon chemoattractant stimulation, proper polarization of neutrophils depends on PA and PLD activity [38]. Moreover, it has been shown that PA accumulates in the pseudopods of RAW264.7 cells engulfing latex beads [10]. These conflicting results showing increased PA in the leading edge of neutrophils or macrophages suggest that lipid signaling may be highly cell-type dependent. The origin of PA may be different (PLD, DGK), as well as the formation of PA and its downstream signaling in unstimulated cells (our results in HeLa cells) compared to latex bead or chemoattractant-stimulated cells, which depend on receptor activation. The time course involved is drastically different as well (tens of minutes to hours in HeLa cells compared to seconds to minutes for neutrophils).

These results are also consistent with the DAG and PA levels found in MSC80 and OLN93 cell lines. PA levels were lower in the processes than in the cell body, whereas DAG showed the opposite profile (Fig. 5). Thus, higher DAG/PA ratios seem to be related to membrane protrusion and process extension. Higher PA levels in the cell body, activating RhoA and myosin II, may stabilize local cytoskeleton and prevent growth of new processes. Consistent with this, inhibition of ROCK or myosin II in oligodendrocytes led to increased branching [39], and was shown to induce multiple minor processes in neurons [40]. Remarkably, conditional knocked-out mice lacking lipin1 (a PA phosphatase) in Schwann cells showed myelination deficiencies [41]. The accumulation of PA in these cells may affect process extension, a prerequisite for proper myelination [33].

## Conclusions

A new FRET probe for PA has been developed and used to analyze fluctuations in the PA pools of the plasma membrane. The biosensor responded to both increases and decreases of PA and displayed wider dynamic range than previously described probes. We also reported PA fluctuations during cell migration, mitosis, and membrane process extension. Our data also suggest that the regulation of PA levels may be more complex than anticipated, exhibiting differences among cell types. This biosensor, which could also be targeted to other membranes, will be very useful to expand our knowledge of the role of PA in biochemical and signaling pathways in live cells.

## Materials and Methods

### Reagents

4-Fluoro-N-(2-(4-(5-fluoro-1H-indol-1-yl)piperidin-1-yl)ethyl) benzamide (FIPI), dioleoyl-phosphatidic acid (DOPA), egg yolk phosphatidylcholine, oleic acid, human epidermal growth factor (EGF), PMA and propranolol were purchased from Sigma-Aldrich.

### Cloning and plasmid preparation

Oligonucleotides used for cloning (Thermo Fisher Scientific) are listed in Table S1. The circularly permuted Venus cloning cassette [16] was a gift from Dr. Atsushi Miyawaki (BSI, RIKEN, Saitama, Japan). Six *yellow cameleon* FRET calcium sensors in vector pRSETB (Invitrogen) [16], differing from each other in the acceptor fluorescent protein (Venus or circularly permuted Venus), were used. For each of the six Venus variants, the following cloning procedure was performed. To insert linker 2, oligonucleotides 1 and 2 were hybridized, 5′ phosphorylated and cloned into the *Sph*I restriction site of the pRSETB vectors (downstream of ECFP). Then, to insert linker 3, oligonucleotides 3 and 4 were hybridized, 5′ phosphorylated and cloned into the SacI restriction site (upstream of Venus or circularly permuted Venus). The resulting constructs were cloned into the BamHI/EcoRI restriction sites of pcDNA3.1 (Invitrogen). A plasma membrane-targeting peptide consisting of amino acids 1–12 of lymphocyte-specific protein tyrosine kinase (Lck) [15] was inserted upstream of ECFP. To this end, oligonucleotides 5 and 6 were hybridized, 5′ phosphorylated and cloned into HindIII/BamHI sites of pcDNA3.1. Next, to insert linker 1 between Lck(1–12) and ECFP, oligonucleotides 7 and 8 were hybridized, 5′ phosphorylated and cloned into the BamHI site. Then, the SphI site downstream of linker 2 and the SacI site upstream of linker 3 were mutated in the whole series of constructs, to generate two BsiWI restriction sites using oligonucleotides 9 and 10. The sequence coding for Spo20 (51–91) was amplified by PCR using as template GFP-Spo20(51–91), kindly provided by Dr. Nicolas Vitale (INCI, Strasbourg, France) [14], and oligonucleotides 11 and 12. The PCR fragments were cloned into the BsiWI restriction sites of the pcDNA3.1 constructs. The construct carrying cpVenus 173 was selected (see Results). The ATG of ECFP was mutated using oligonucleotide 13 to ensure that translation initiation occurred only at the Lck initiation codon. The resulting construct was named pmPAS (*p*lasma *m*embrane *PA S*ensor) (Fig. 1A). Size and integrity of pmPAS in transfected cells were confirmed (Fig. 1C). Restriction enzyme digestions, ligations (T4 ligase), phosphorylations (polynucleotide kinase), dephosphorylations (shrimp alkaline phosphatase) and PCRs (Pfu polymerase) were performed according to the manufacturer's protocols. All enzymes were from Fermentas, except for T4 ligase, which was from Promega. Mutagenesis was performed with the QuikChange Lightning Multi Site-Directed Mutagenesis Kit (Agilent Technologies). The constructs were screened with PCR and restriction enzyme digestions. Sequencing was performed by Macrogen (South Korea). The plasmid Daglas-pm1, a FRET sensor for DAG, was a gift from Prof. Yoshio Umezawa (University of Tokyo, Tokyo, Japan). Plasmids coding for PLD1 and PLD2 were a gift from Dr. Michael Frohman (State University of New York, Stony Brook, USA) [42].

### Cell Culture and Transfection

HeLa, HT29 [43], and HCT116 [44] cell lines were cultured in Dulbecco’s modified Eagle medium (DMEM, Gibco), supplemented with 10% fetal calf serum, 2 mM L-glutamine, 100 U/mL penicillin and 100 µg/mL streptomycin sulfate (all reagents from Lonza) at 37°C with 5% CO_2_ in a humidified atmosphere. For migration experiments, HeLa cells were cultured in supplemented Alpha modified Eagle medium (α-MEM, Lonza). MSC80 [45] and OLN93 [46] cell lines were cultured in the same conditions as the cells mentioned above, but culture dishes were pre-coated with poly-D-lysine (Sigma-Aldrich). HeLa, HT29 and HCT116 cells were transfected with Lipofectamine 2000 (Invitrogen), and MSC80 and OLN93 cells were transfected with jetPRIME (Polyplus Transfection) according to the manufacturer's protocols.

### Cell imaging

Cells were imaged in glass bottom dishes (ibidi GmbH) 24–48 hours after transfection. Culture medium was replaced with Hanks balanced salt solution (Gibco) supplemented with 5.55 mM D-glucose and 10 mM Hepes, pH 7.4. Chemical stimuli were diluted directly into the Hank’s balanced salt solution in the dish. Alternatively, a gravity driven flow-perfusion system coupled to a vacuum pump for output was used to exchange solutions. For migration experiments, cells were imaged in supplemented α-MEM, and a PeCon system was used to keep the whole microscope chamber at 37°C and to sustain a constant flow of humidified air with 5% CO_2_ in the dish chamber.

Fluorescence imaging of cells was performed using an epifluorescence inverted microscope (DMIRE-2, Leica) with PlanApo 40x (N.A. 1.25) or PlanApo 63x (N.A. 1.4) oil immersion objectives. The excitation light source was a high speed scanning polychromator with Xe lamp (C7773, Hamamatsu Photonics), using the 10 nm slit. The emission filter wheel was controlled by a Lambda-10 device (Sutter Instruments). Images were acquired with an EM-CCD camera (C9100-13) and Aquacosmos 2.6 software was used to control all devices (both from Hamamatsu Photonics). In FRET experiments, three different images were sequentially taken at each time point: the ECFP image was obtained by exciting ECFP (430 nm) and monitoring its emission (475/20 nm), the Venus image was acquired by exciting Venus fluorescent protein (500 nm) and monitoring its emission (535/22 nm), and the FRET image was obtained by exciting the donor ECFP (430 nm), and monitoring the emission of the acceptor Venus (535/22 nm). Filter specifications are detailed in Table S2. ImageJ software [47] with customized macros was used to subtract the background from raw images and to create ratio images in intensity-modulated display mode (Venus images were used as intensity modulator). We also used the so called 3-Cube method to estimate the absolute FRET efficiency (*E*) and the relative concentration of donor and acceptor fluorochromes ([D]/[A]) [48], [49].

Membrane localization of pmPAS in HeLa cells was confirmed in a Leica TCS SP2 AOBS confocal module equipped with a Plan Apo 63x (N.A 1.32) oil immersion objective. The Venus fluorescent protein of the chimera was excited with an Argon laser at 488 nm.

### Liposome preparation

Liposome preparation was adapted from [50]. For oleic acid liposomes (lipoOA), oleic acid was mixed with a chloroform:methanol solution (1∶1) containing an equal amount of phosphatidylcholine. For dioleoyl-phosphatidic acid liposomes (lipoDOPA), aliquots of 5 mg/mL dioleoyl-phosphatidic acid dissolved in chloroform:methanol (1∶1) were prepared. These solutions were dried under vacuum and the resulting films were hydrated in 150 µL of liposome resuspending buffer (10 mM TrisHCl, 100 mM NaCl, pH 7.4) for 30 minutes at 37°C. The suspensions were pipetted up and down several times and subjected to 3 cycles of 1 min vortexing at maximum speed. The resulting milky suspensions containing mostly multilamellar vesicles were transferred to Falcon tubes containing 450 µL of Hank’s balanced salt solution. Liposomes were generated using an UP200s sonicator probe (Dr. Hielscher GmbH). Nine cycles of 20 pulses (30% amplitude) of 0.5 s each were applied with a 20 s pause between them. Tubes were kept on ice during sonication to prevent overheating and subjected to a short spindown every 3 cycles. Liposomes were added to the cells immediately after preparation.

### Sucrose density gradient fractionation

This protocol was adapted from [51], [52]. Briefly, confluent transfected HeLa cells were washed with ice-cold PBS, scraped, collected in 1 mL of lysis buffer (10 mM Tris, pH 7.4, 150 mM NaCl, 5 mM EDTA, 2 mM PMSF, 20 µg/ml leupeptin, and 20 µg/ml aprotinin, 1% Triton X100) and left on ice for 1 h. Sucrose solutions (80%, 35% and 5%, w/v) were prepared in lysis buffer lacking detergent and protease inhibitors. The lysate was centrifuged (700 ***g***, 10 min, 4°C), the post-nuclear supernatant was diluted 1∶1 with 80% sucrose solution and placed at the bottom of an ultracentrifuge tube. The latter was layered with 4 mL of 35% sucrose, 1 mL of 5% sucrose and 5.5 mL of lysis buffer without detergent or protease inhibitors. Tubes were centrifuged at 260,600 ***g*** for 18 h at 4°C (SW41Ti rotor, Beckman). After centrifugation, the top 4.5 mL were discarded and nine equal volume fractions starting from the top were collected. Fractions were analyzed by Western and dot blotting.

### SDS-PAGE, Western blotting and dot blotting

To check the size and integrity of the expressed biosensors, cells were lysed and kept on ice for 1 h with periodic mixing. Cell lysates were centrifuged at 700 ***g*** at 4°C for 10 minutes. Next, 20 µL of the lysates or 30 µL of ultracentrifugation fractions were run in 10% SDS-PAGE gels. Proteins were transferred to PVDF membranes (Amersham Biosciences) using a semidry method (BioRad). Membranes were blocked with 5% non-fat milk in TBS with 0.1% Tween20 and probed with anti-GFP antibody (Covance, 1∶1,000) overnight at 4°C. For dot-blot analysis, 2 µL of each ultracentrifugation fraction were dot-blotted on nitrocellulose membranes. The membranes were blocked as indicated above and probed for Caveolin-1 (Abcam, 1∶1,000). Immunoblots were then probed with horseradish peroxidase conjugated secondary antibodies (1∶1000) for 1 h at room temperature. Finally, blots were incubated with ECL Super Signal West Dura Extended Duration Substrate (Thermo Scientific) and chemiluminescence was imaged with a FujiFILM LAS-3000 CCD camera.

## Supporting Information

Figure S1 **PA metabolic network.** Enzymes related with PA metabolism are shown in blue italics. Enzyme activators (green) and inhibitors (red) used in this study are denoted by “+” or “−“, respectively. Arrows indicate the direction of reactions. Glyc3P, glycerol-3-phosphate. FA-CoA, fatty acid-coenzyme A. PC, phosphatidylcholine. PA, phosphatidic acid. LPA, lysophophatidic acid. DAG, diacylglycerol. TAG, triacylglycerol. PE, phosphatidylethanolamine. PS, phosphatidylserine. CDP-DAG, cytidine diphosphate diacylglycerol. PI, phosphatidylinositol. PI4P, phosphatidylinositol-4-phosphate. PI(4,5)P_2_, phosphatidylinositol-4,5-bisphosphate. PI(3,4,5)P_3_, phosphatidylinositol-3,4,5-trisphosphate. IP_3_, inositol trisphosphate. PLD, phospholipase D. LPAAT, lysophosphatidic acid acyltransferase. PLA_2_, phospholipase A_2_. mitoPLD, mitochondrial phospholipase D. DGK, diacylglycerol kinase. PAP, PA phosphatase. CDP-DAGS, CDP-DAG synthetase. PIK1, phosphatidylinositol-4-kinase 1. PI5K, phosphatidylinositol-5-kinase. PI3K, phosphatidylinositol-3-kinase. 5-P’ase, 5-phosphatase. PTEN, phosphatase and tensin homolog. PLC, phospholipase C.
(TIF)

Figure S2 **Characterization of chimeras targeted to the plasma membrane with Venus (or variants of circularly permuted Venus) as FRET acceptor fluorescent protein in HeLa cells.** (**A**) Basal ECFP/FRET value of the different chimeras. (**B**) Normalized ECFP/FRET of cells expressing the chimeras, challenged with 100 µM propranolol and 100 nM PMA (*n* ranges from 6 to 12 cells. Error bars were omitted for better comparison). (**C**) Cells expressing constructs containing one (*n* = 12, 4 independent experiments) or two copies of Spo20 (51–91) (*n* = 5, 2 independent experiments) as PABD were challenged with 100 µM propranolol and 100 nM PMA. Error bars indicate the mean±SEM. (**D**) Confocal image (with orthogonal views) of a HeLa cell expressing pmPAS (Venus channel). The scale bar indicates 20 µm.
(TIF)

Figure S3 **Image processing, estimation of donor to acceptor expression ratio ([D]/[A]) and FRET efficiency (** ***E*** **) of HeLa cells expressing pmPAS.** (**A**) ECFP, FRET and Venus fluorescence images. (**B**) ECFP/FRET ratio images, donor to acceptor relative concentration ([D]/[A]) images and FRET efficiency (*E*) images of HeLa cells expressing pmPAS before and 5 min after addition of 100 µM propranolol and 100 nM PMA. The 3-cube method was used to calculate [D]/[A] images and FRET efficiency images (see Materials and Methods). The scale bar indicates 20 µm.
(TIF)

Figure S4 **Translocation of GFP-Spo20 to the plasma membrane in response to PMA.** (**A**) A representative HeLa cell expressing GFP-Spo20, before and 30 min after addition of PMA (100 nM). The scale bar indicates 20 µm. (**B**) Time course of normalized fluorescence (intensity of cytoplasmic and membrane area divided by intensity over the nucleus) of HeLa cells transfected with GFP-Spo20 challenged with PMA as in (A), with or without preincubation with FIPI (1 µM, 30 min) (*n* = 5 and 3 independent experiments for each condition). Error bars indicate the mean±SEM.
(TIF)

Figure S5 **Representative responses of HeLa cells upon addition of liposomes containing dioleoyl PA (lipoDOPA, 200 µM).** (**A**) and (**B**) Venus intensity and ECFP/FRET ratio images (top panels) and normalized ECFP/FRET time course (bottom panels) of cells expressing pmPAS. The ECFP/FRET ratio in the images was coded according to the indicated pseudocolor scale. (**C**) Images and intensity time course of a cell expressing GFP-Spo20. (**A–C**) The time courses and magnified images refer to the area indicated by a box in the corresponding gray images. Images were acquired at the indicated time (m, minutes) before or after lipoDOPA addition to the cells under the microscope. The scale bars indicate 20 µm.
(TIF)

Figure S6 **Representative responses of HeLa cells expressing pmPAS upon addition of liposomes containing oleic acid (lipoOA, 500 µM).** (**A–D**) Venus intensity images (C and D), ECFP/FRET ratio images and normalized ECFP/FRET time course of cells expressing pmPAS. In (**D**), a cell was sequentially challenged with lipoDOPA (200 µM) and lipoOA. Images were acquired at the indicated time (m, minutes) before or after liposome addition. The time courses and magnified images refer to the area shown by a box in the corresponding images. The ECFP/FRET ratio was coded according to the indicated pseudocolor scale. The scale bars indicate 20 µm.
(TIF)

Figure S7 **Effect of lysophosphatidic acid (LPA) in MSC80 cells expressing pmPAS.** (**A–C**) Pseudocolor ECFP/FRET images of MSC80 cells expressing pmPAS challenged with liposomes containing LPA (lipoLPA, 200 uM) and phosphatidylcholine (**A**), control liposomes containing phosphatidylcholine alone (lipoPC) (**B**), or LPA without the use of liposomes (LPA, 200 uM) (**C**). Scale bars represent 20 µm and the ECFP/FRET images were coded according to the indicated pseudocolor scale. (**D**) Time course of normalized ECFP/FRET values of cells stimulated as in (A-C). Error bars represent the mean±SEM. LipoLPA *n* = 9, lipoPC *n* = 14, LPA *n* = 9 cells.
(TIF)

Figure S8 **Representative HT29 and HCT116 cells in culture expressing pmPAS.** HT29 cells showed a more rounded shape and lower adherence to the surface compared to HCT116 cells. The scale bars indicate 20 µm.
(TIF)

Table S1 **Oligonucleotides used for cloning and for mutagenesis (the latter are labelled** ***mut*** **).**
(DOCX)

Table S2 **Imaging channels defined by different excitation and emission wavelength combinations.**
(DOCX)

Video S1 **ECFP/FRET video of HeLa cells expressing pmPAS sequentially challenged with propranolol (100 µM) and PMA (100 nM).** The scale bar indicates 20 µm. The ECFP/FRET ratio was coded according to the indicated pseudocolor scale.
(AVI)

Video S2 **ECFP/FRET video of a region of a HeLa cell expressing pmPAS during addition of dioleoyl PA liposomes (lipoDOPA).** The scale bar indicates 20 µm. The ECFP/FRET ratio was coded according to the indicated pseudocolor scale.
(AVI)

Video S3 **Venus intensity and ECFP/FRET video of a HeLa cell expressing pmPAS during incubation with oleic acid/phosphatidylcholine liposomes (lipoOA).** The intensity of Venus was adjusted to show filopodia and thus appears mostly saturated. The scale bar indicates 20 µm. The ECFP/FRET ratio was coded according to the indicated pseudocolor scale.
(AVI)

Video S4 **ECFP/FRET video of a HeLa cell expressing pmPAS during migration.** The assay was run at 37°C with a humidified 5% CO_2_ atmosphere in fully supplemented culture medium. The scale bar indicates 20 µm. The ECFP/FRET ratio was coded according to the indicated pseudocolor scale. The video represents 3 h 40min of the assay (images were taken every 5 min).
(AVI)

Video S5 **ECFP/FRET video of a HeLa cell expressing pmPAS during migration.** The assay was run at 37°C with a humidified 5% CO_2_ atmosphere in fully supplemented medium. The scale bar indicates 20 µm. The ECFP/FRET ratio was coded according to the indicated pseudocolor scale.
(AVI)
