# Supplementary figures and images for: Visualization of Phosphatidic Acid Fluctuations in the Plasma Membrane of Living Cells
Source: PLoS One. 2014 Jul 15;9(7):e102526. doi: 10.1371/journal.pone.0102526 (PMC4099201; doi:10.1371/journal.pone.0102526)

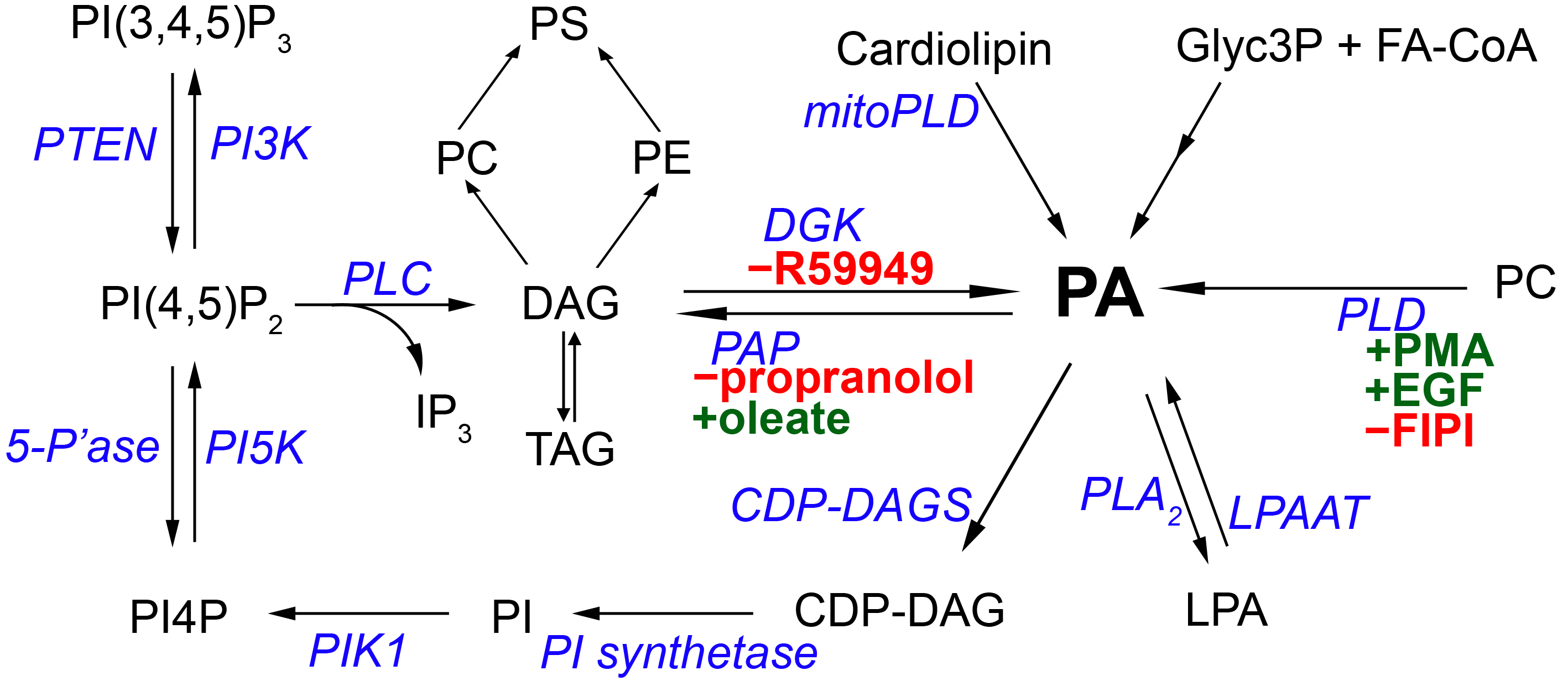

Supplement: Figure S1 — PA metabolic network. Enzymes related with PA metabolism are shown in blue italics. Enzyme activators (green) and inhibitors (red) used in this study are denoted by “+” or “−“, respectively. Arrows indicate the direction of reactions. Glyc3P, glycerol-3-phosphate. FA-CoA, fatty acid-coenzyme A. PC, phosphatidylcholine. PA, phosphatidic acid. LPA, lysophophatidic acid. DAG, diacylglycerol. TAG, triacylglycerol. PE, phosphatidylethanolamine. PS, phosphatidylserine. CDP-DAG, cytidine diphosphate diacylglycerol. PI, phosphatidylinositol. PI4P, phosphatidylinositol-4-phosphate. PI(4,5)P2, phosphatidylinositol-4,5-bisphosphate. PI(3,4,5)P3, phosphatidylinositol-3,4,5-trisphosphate. IP3, inositol trisphosphate. PLD, phospholipase D. LPAAT, lysophosphatidic acid acyltransferase. PLA2, phospholipase A2. mitoPLD, mitochondrial phospholipase D. DGK, diacylglycerol kinase. PAP, PA phosphatase. CDP-DAGS, CDP-DAG synthetase. PIK1, phosphatidylinositol-4-kinase 1. PI5K, phosphatidylinositol-5-kinase. PI3K, phosphatidylinositol-3-kinase. 5-P’ase, 5-phosphatase. PTEN, phosphatase and tensin homolog. PLC, phospholipase C. (TIF) [file pone.0102526.s001.tif]

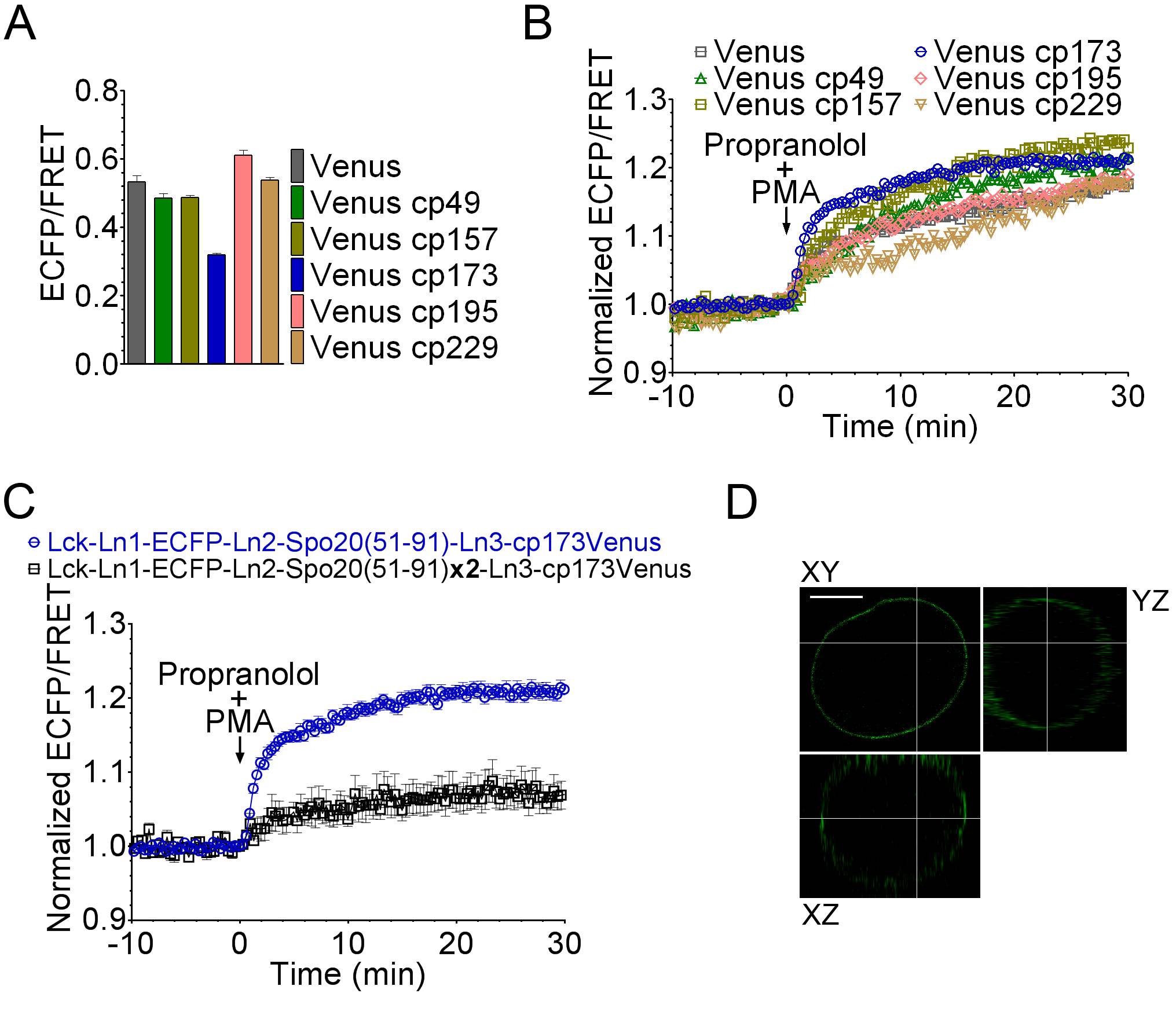

Supplement: Figure S2 — Characterization of chimeras targeted to the plasma membrane with Venus (or variants of circularly permuted Venus) as FRET acceptor fluorescent protein in HeLa cells. (A) Basal ECFP/FRET value of the different chimeras. (B) Normalized ECFP/FRET of cells expressing the chimeras, challenged with 100 µM propranolol and 100 nM PMA (n ranges from 6 to 12 cells. Error bars were omitted for better comparison). (C) Cells expressing constructs containing one (n = 12, 4 independent experiments) or two copies of Spo20 (51–91) (n = 5, 2 independent experiments) as PABD were challenged with 100 µM propranolol and 100 nM PMA. Error bars indicate the mean±SEM. (D) Confocal image (with orthogonal views) of a HeLa cell expressing pmPAS (Venus channel). The scale bar indicates 20 µm. (TIF) [file pone.0102526.s002.tif]

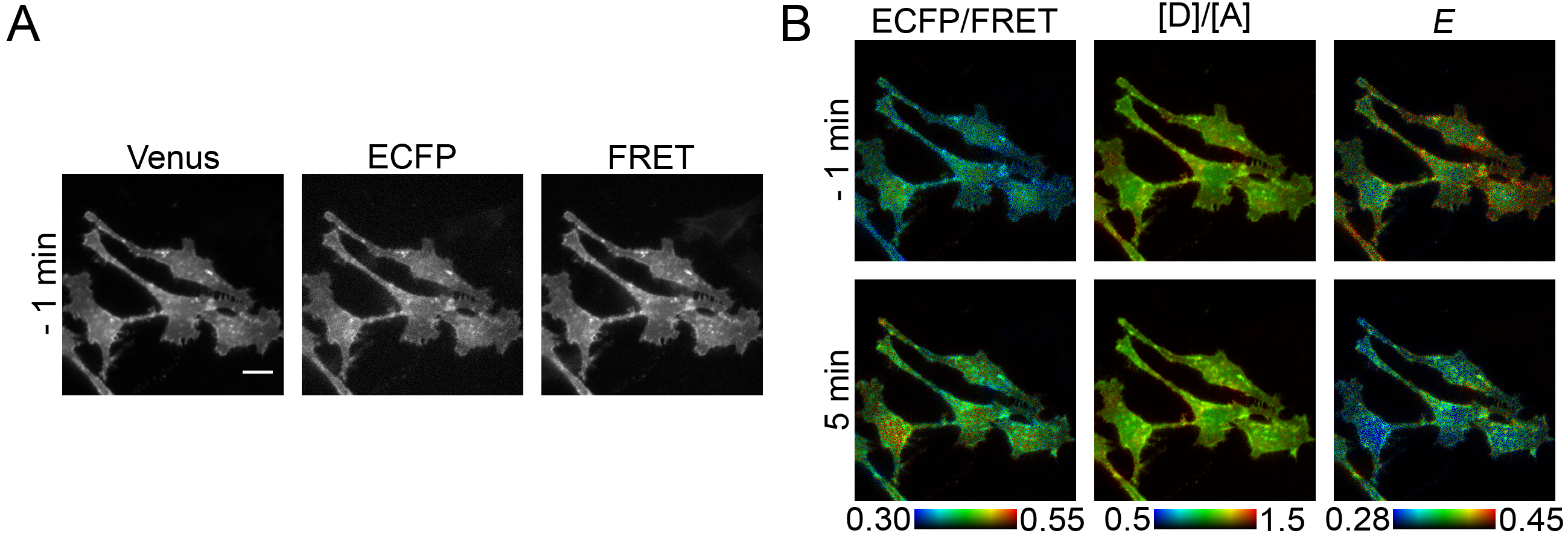

Supplement: Figure S3 — Image processing, estimation of donor to acceptor expression ratio ([D]/[A]) and FRET efficiency ( E ) of HeLa cells expressing pmPAS. (A) ECFP, FRET and Venus fluorescence images. (B) ECFP/FRET ratio images, donor to acceptor relative concentration ([D]/[A]) images and FRET efficiency (E) images of HeLa cells expressing pmPAS before and 5 min after addition of 100 µM propranolol and 100 nM PMA. The 3-cube method was used to calculate [D]/[A] images and FRET efficiency images (see Materials and Methods). The scale bar indicates 20 µm. (TIF) [file pone.0102526.s003.tif]

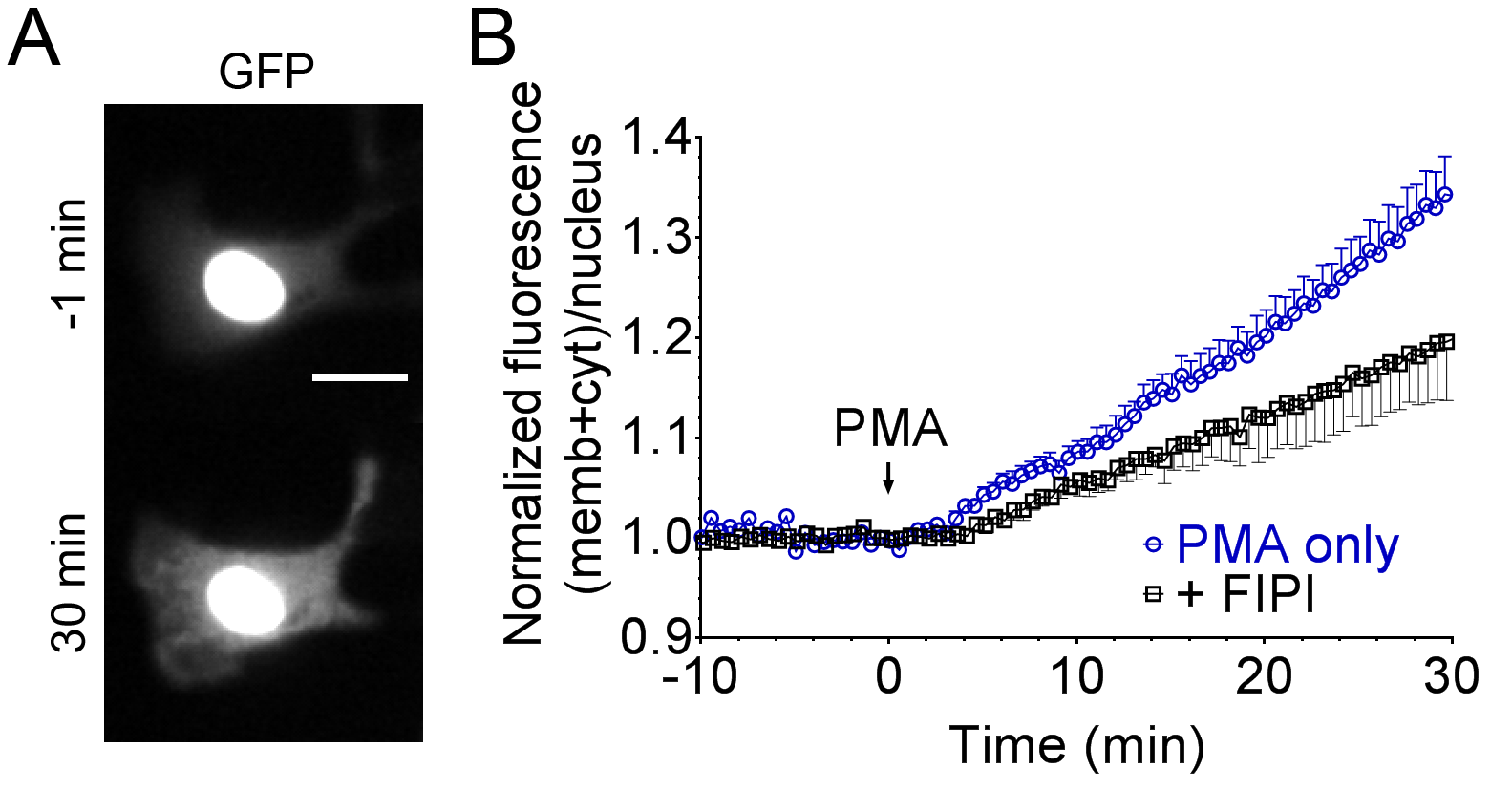

Supplement: Figure S4 — Translocation of GFP-Spo20 to the plasma membrane in response to PMA. (A) A representative HeLa cell expressing GFP-Spo20, before and 30 min after addition of PMA (100 nM). The scale bar indicates 20 µm. (B) Time course of normalized fluorescence (intensity of cytoplasmic and membrane area divided by intensity over the nucleus) of HeLa cells transfected with GFP-Spo20 challenged with PMA as in (A), with or without preincubation with FIPI (1 µM, 30 min) (n = 5 and 3 independent experiments for each condition). Error bars indicate the mean±SEM. (TIF) [file pone.0102526.s004.tif]

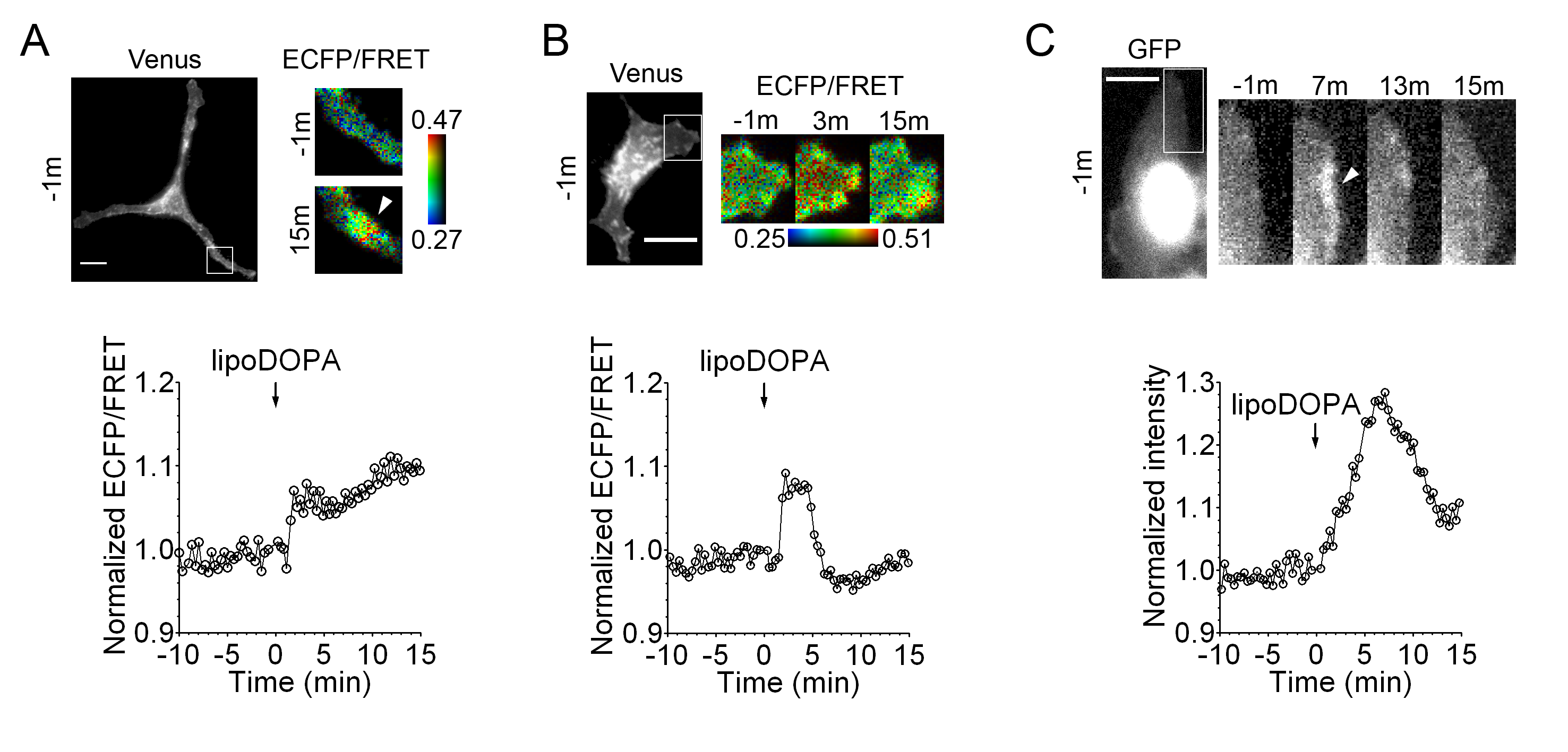

Supplement: Figure S5 — Representative responses of HeLa cells upon addition of liposomes containing dioleoyl PA (lipoDOPA, 200 µM). (A) and (B) Venus intensity and ECFP/FRET ratio images (top panels) and normalized ECFP/FRET time course (bottom panels) of cells expressing pmPAS. The ECFP/FRET ratio in the images was coded according to the indicated pseudocolor scale. (C) Images and intensity time course of a cell expressing GFP-Spo20. (A–C) The time courses and magnified images refer to the area indicated by a box in the corresponding gray images. Images were acquired at the indicated time (m, minutes) before or after lipoDOPA addition to the cells under the microscope. The scale bars indicate 20 µm. (TIF) [file pone.0102526.s005.tif]

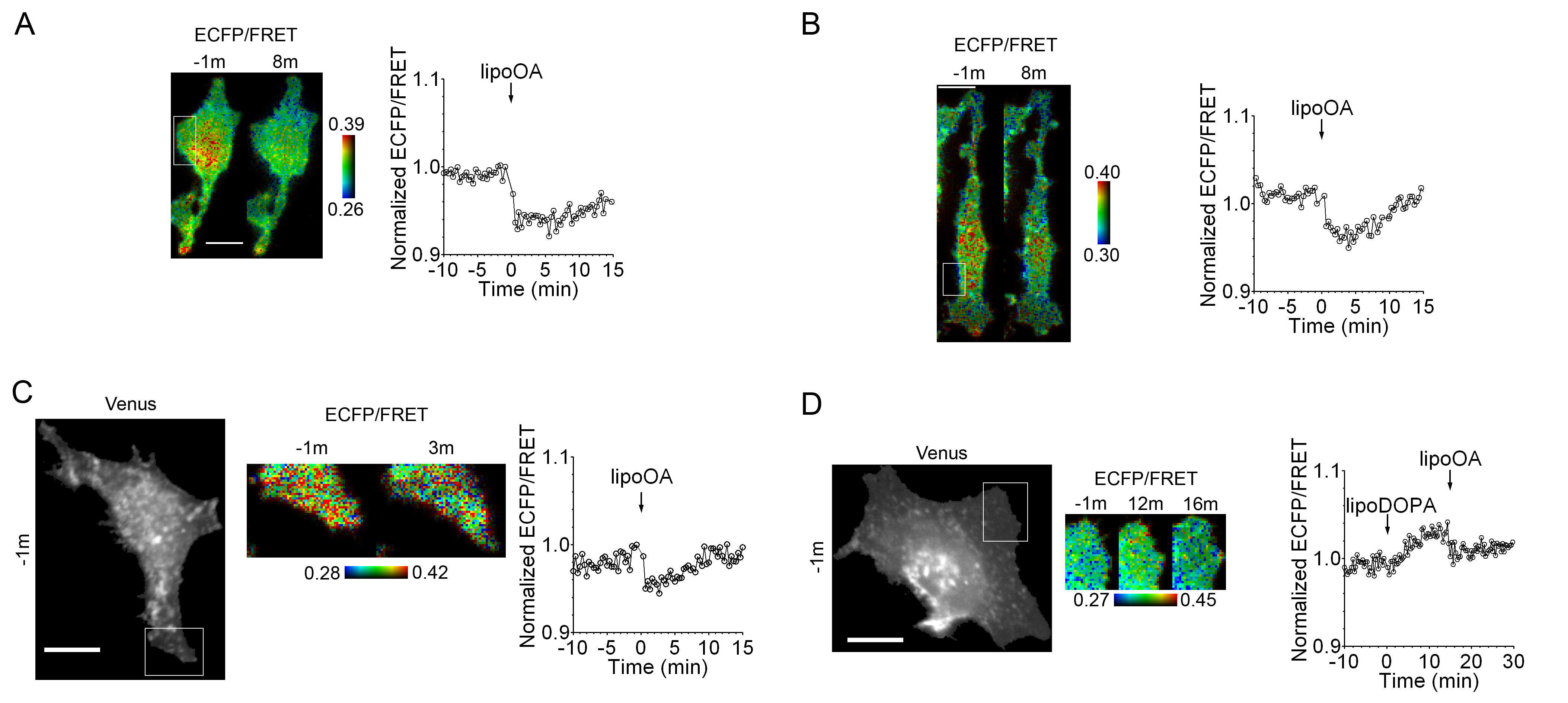

Supplement: Figure S6 — Representative responses of HeLa cells expressing pmPAS upon addition of liposomes containing oleic acid (lipoOA, 500 µM). (A–D) Venus intensity images (C and D), ECFP/FRET ratio images and normalized ECFP/FRET time course of cells expressing pmPAS. In (D), a cell was sequentially challenged with lipoDOPA (200 µM) and lipoOA. Images were acquired at the indicated time (m, minutes) before or after liposome addition. The time courses and magnified images refer to the area shown by a box in the corresponding images. The ECFP/FRET ratio was coded according to the indicated pseudocolor scale. The scale bars indicate 20 µm. (TIF) [file pone.0102526.s006.tif]

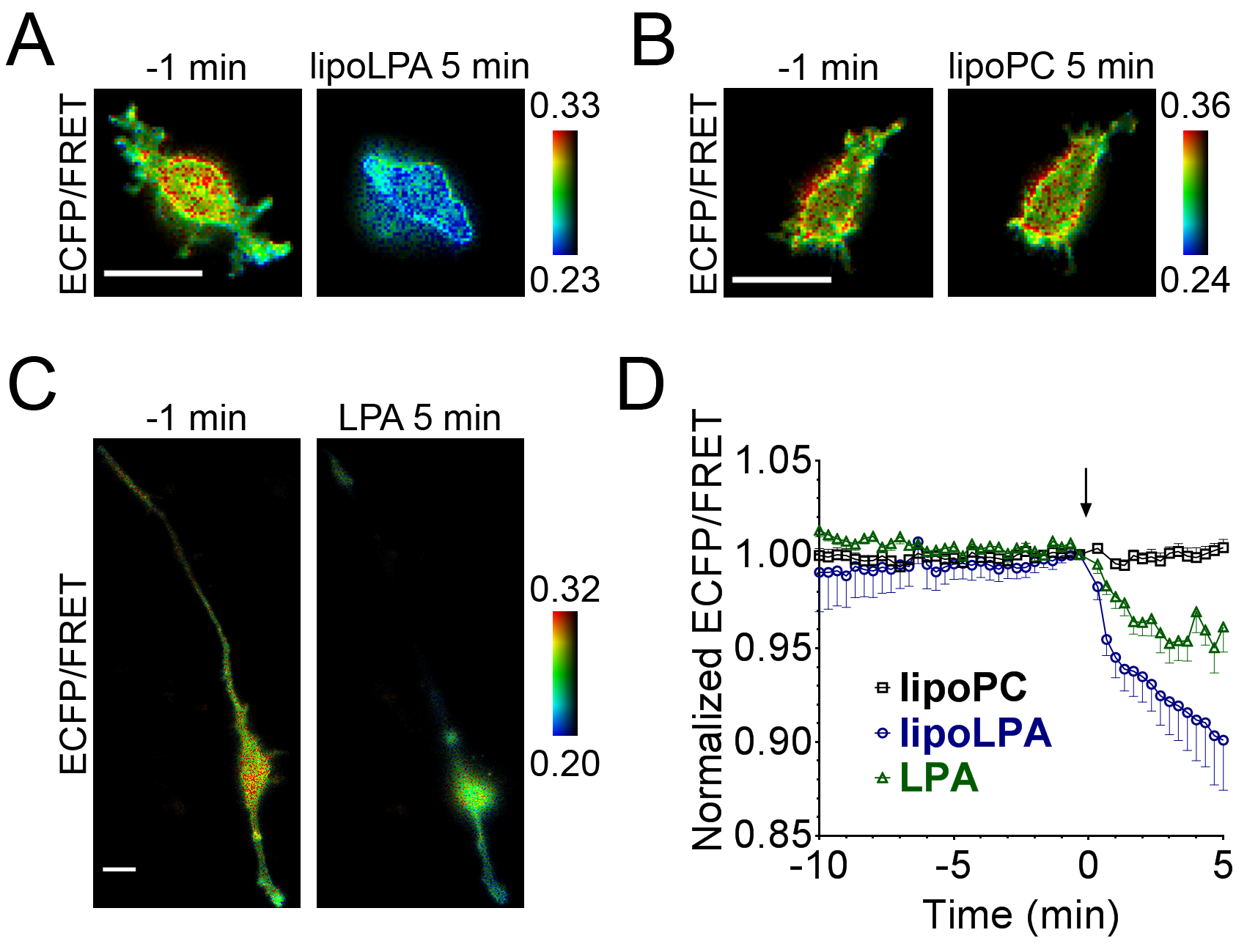

Supplement: Figure S7 — Effect of lysophosphatidic acid (LPA) in MSC80 cells expressing pmPAS. (A–C) Pseudocolor ECFP/FRET images of MSC80 cells expressing pmPAS challenged with liposomes containing LPA (lipoLPA, 200 uM) and phosphatidylcholine (A), control liposomes containing phosphatidylcholine alone (lipoPC) (B), or LPA without the use of liposomes (LPA, 200 uM) (C). Scale bars represent 20 µm and the ECFP/FRET images were coded according to the indicated pseudocolor scale. (D) Time course of normalized ECFP/FRET values of cells stimulated as in (A-C). Error bars represent the mean±SEM. LipoLPA n = 9, lipoPC n = 14, LPA n = 9 cells. (TIF) [file pone.0102526.s007.tif]

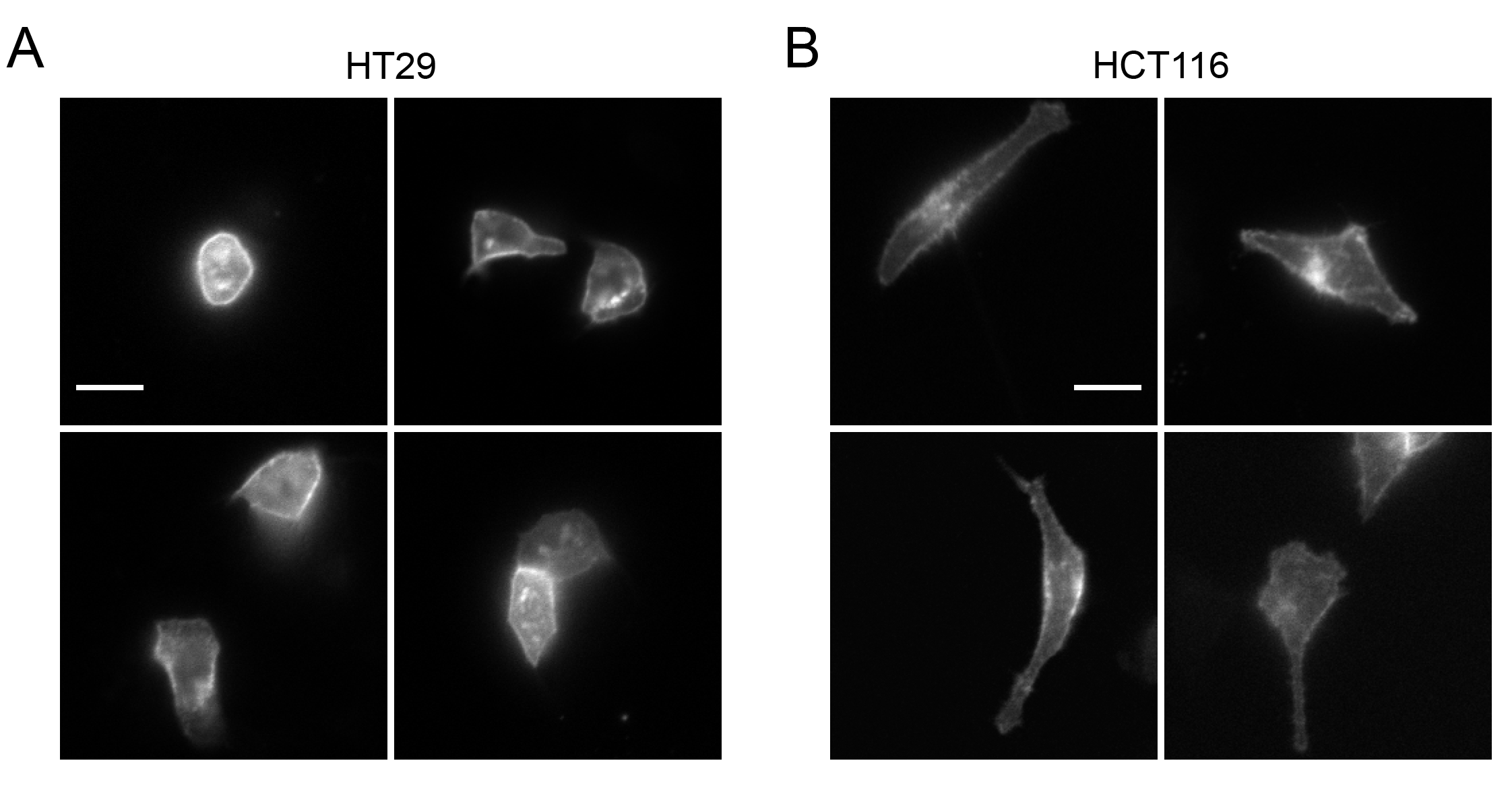

Supplement: Figure S8 — Representative HT29 and HCT116 cells in culture expressing pmPAS. HT29 cells showed a more rounded shape and lower adherence to the surface compared to HCT116 cells. The scale bars indicate 20 µm. (TIF) [file pone.0102526.s008.tif]
