# Supplementary material for: Visualization of Phosphatidic Acid Fluctuations in the Plasma Membrane of Living Cells
Source: PLoS One. 2014 Jul 15;9(7):e102526. doi: 10.1371/journal.pone.0102526 (PMC4099201; doi:10.1371/journal.pone.0102526)
Supplement: Table S1 — Oligonucleotides used for cloning and for mutagenesis (the latter are labelled mut ). (DOCX) [file pone.0102526.s009.docx]

Table S1. Oligonucleotides used for cloning and for mutagenesis (the latter are labelled *mut*).

| ***Number*** | ***Name*** | ***Sequence (5’>3’)*** |
| --- | --- | --- |
| 1 | SphI_Ln2_SphI_FW | CGGGAAGCAGCAGCAAGAGAAGCAGCAGCAAGAGAAGCAGCAGCAAGAGGCGGCGAAGCAGCAGCAAGAGAAGCAGCAGCAAGAGAAGCAGCAGCAAGACGCATG |
| 2 | SphI_Ln2_SphI_REV | CGTCTTGCTGCTGCTTCTCTTGCTGCTGCTTCTCTTGCTGCTGCTTCGCCGCCTCTTGCTGCTGCTTCTCTTGCTGCTGCTTCTCTTGCTGCTGCTTCCCGCATG |
| 3 | SacI_Ln3_SacI_FW | CGAAGCAGCAGCAAGAGAAGCAGCAGCAAGAGAAGCAGCAGCAAGAGAAGCAGCAGCAAGAGAAGCAGCAGCAAGAGAAGCAGCAGCAAGAGAGCT |
| 4 | SacI_Ln3_SacI_REV | CTCTTGCTGCTGCTTCTCTTGCTGCTGCTTCTCTTGCTGCTGCTTCTCTTGCTGCTGCTTCTCTTGCTGCTGCTTCTCTTGCTGCTGCTTCGAGCT |
| 5 | HIndIII_Lck(1-12)_BamHI_FW | AGCTTACCATGGGCTGCGGGTGTAGTTCTCATCCGGAAGACGACGCG |
| 6 | HIndIII_Lck(1-12)_BamHI_REV | GATCCGCGTCGTCTTCCGGATGAGAACTACACCCGCAGCCCATGGTA |
| 7 | BamHI_Ln1_BamHI_FW | GATCCCGAAGCAGCAGCAAGAGAAGCAGCAGCAAGAGAAGCAGCAGCAAGAGAAGCAGCAGCAAGAGAAGCAGCAGCAAGAGAAGCAGCAGCAAGAGAAGCAGCAGCAAGAGCG |
| 8 | BamHI_Ln1_BamHI_REV | GATCCGCTCTTGCTGCTGCTTCTCTTGCTGCTGCTTCTCTTGCTGCTGCTTCTCTTGCTGCTGCTTCTCTTGCTGCTGCTTCTCTTGCTGCTGCTTCTCTTGCTGCTGCTTCGG |
| 9 | Ln2_SphI>BsiWI (mut) | CAGCAGCAAGACGTACGCGGGACTGCTG |
| 10 | SacI>BsiWI_Ln3 (mut) | CCCAGCGGAGATGCGTACGGAAGCAGCAGCAAGAG |
| 11 | BsiWI_Spo20(51-91)_BsiWI_FW | ACATTCGTACGGGCGGATCTATGGACAATTGTTCAGG |
| 12 | BsiWI_Spo20(51-91)_BsiWI_REV | CTATTCGTACGAGATCCTCCACTAGTCTTAGTGGCG |
| 13 | ECFP_M1V (mut) | CAAGAGCGGATCCCGTGGTGAGCAAGG |
