# Supplementary material for: Visualization of Phosphatidic Acid Fluctuations in the Plasma Membrane of Living Cells
Source: PLoS One. 2014 Jul 15;9(7):e102526. doi: 10.1371/journal.pone.0102526 (PMC4099201; doi:10.1371/journal.pone.0102526)
Supplement: Table S2 — Imaging channels defined by different excitation and emission wavelength combinations. (DOCX) [file pone.0102526.s010.docx]

Table S2. Imaging channels defined by different excitation and emission wavelength combinations.

| ***Imaging channels*** | ***Monochromator excitation*** | ***Excitation filter*** | ***Dichroic mirror*** | ***Emission filter*** |
| --- | --- | --- | --- | --- |
| **ECFP** | 430 | 422/30 ^a^ | **444**/520/590 ^b^ | 475/20 ^c^ |
| **Venus** | 500 | 503/18 ^a^ | 444/**520**/590 ^b^ | 535/22 ^d^ |
| **FRET** | 430 | 422/30 ^a^ | **444**/520/590 ^b^ | 535/22 ^d^ |
| **GFP** | 500 | 503/18 ^a^ | 444/**520**/590 ^b^ | 535/22 ^d^ |

a, BrightLine triple-band bandpass excitation filter (Semrock).

b, BrightLine triple-edge dichroic beamsplitter (Semrock).

c,d, BrightLine single-band bandpass emission filters (Semrock).
